# Defining candidate mRNA and protein EV biomarkers to discriminate ccRCC and pRCC from non-malignant renal cells in vitro

**DOI:** 10.1007/s12032-021-01554-2

**Published:** 2021-07-31

**Authors:** Richard C. Zieren, Liang Dong, David J. Clark, Morgan D. Kuczler, Kengo Horie, Leandro Ferreira Moreno, Tung-Shing M. Lih, Michael Schnaubelt, Louis Vermeulen, Hui Zhang, Theo M. de Reijke, Kenneth J. Pienta, Sarah R. Amend

**Affiliations:** 1 The Brady Urological Institute, Johns Hopkins University School of Medicine, Baltimore, MD 21287 USA; 2 Department of Urology, Amsterdam UMC, University of Amsterdam, Amsterdam, The Netherlands; 3 Department of Urology, Renji Hospital, Shanghai Jiao Tong University School of Medicine, Shanghai, China; 4 Department of Pathology, The Johns Hopkins University, Baltimore, MD 21287 USA; 5 Oncode Institute, Amsterdam UMC, Amsterdam, The Netherlands

**Keywords:** Kidney cancer, Extracellular vesicles, Biomarkers, Multi-omics, EV cargo

## Abstract

Renal cell carcinoma (RCC) accounts for over 400,000 new cases and 175,000 deaths annually. Diagnostic RCC biomarkers may prevent overtreatment in patients with early disease. Extracellular vesicles (EVs) are a promising source of RCC biomarkers because EVs carry proteins and messenger RNA (mRNA) among other biomolecules. We aimed to identify biomarkers and assess biological functions of EV cargo from clear cell RCC (ccRCC), papillary RCC (pRCC), and benign kidney cell lines. EVs were enriched from conditioned cell media by size exclusion chromatography. The EV proteome was assessed using Tandem Mass Tag mass spectrometry (TMT-MS) and NanoString nCounter technology was used to profile 770 cancer-related mRNA present in EVs. The heterogeneity of protein and mRNA abundance and identification highlighted the heterogeneity of EV cargo, even between cell lines of a similar pathological group (e.g., ccRCC or pRCC). Overall, 1726 proteins were quantified across all EV samples, including 181 proteins that were detected in all samples. In the targeted profiling of mRNA by NanoString, 461 mRNAs were detected in EVs from at least one cell line, including 159 that were present in EVs from all cell lines. In addition to a shared EV cargo signature, pRCC, ccRCC, and/or benign renal cell lines also showed unique signatures. Using this multi-omics approach, we identified 34 protein candidate pRCC EV biomarkers and 20 protein and 8 mRNA candidate ccRCC EV biomarkers for clinical validation.

**Supplementary Information:**

The online version contains supplementary material available at 10.1007/s12032-021-01554-2.

## Introduction

Renal cell carcinoma (RCC) is a lethal disease which accounts for more than 400,000 new cases worldwide and over 175,000 deaths annually [1]. A growing number of kidney cancer patients are diagnosed at an early stage. The incidence of localized RCC increased > 2.3-fold between the years 2000 and 2017, compared with 1.6-fold for regional metastatic and 1.3-fold for distant metastatic RCC [2]. This shift is attributed to the discovery of incidental renal masses through the increased use of imaging modalities for other symptoms [3]. This trend may lead to overtreatment, especially in small renal masses (SRM) [4, 5]. The most common (~ 75%) histopathological subtype of RCC is clear cell RCC (ccRCC) followed by papillary RCC (pRCC, ~ 15%). The subtypes and their genetic subtypes (e.g., papillary type 1 and 2) vary in lethality and biological tumorigenesis, resulting in various response to targeted therapies [6, 7]. Paradoxically to the challenges that come with early diagnosis, still one in every six patients presents with distant metastatic disease at time of diagnosis [8]. Thus, complementary diagnostic and prognostic RCC biomarkers, while not currently in clinical use, would play an important role to stratify patients with aggressive tumors in urgent need of treatment, as well as prevent unnecessary surgery and local treatment in patients with benign SRM.

Extracellular vesicles (EVs) are nanoparticles (50–1000 nm) that are secreted by all living cells, including cancer cells [9] and their bilipid membrane protects molecular cargo including RNA and protein [10]. EVs can be obtained non-invasively from biofluids such as urine and plasma as well as from cell-conditioned media (CCM) [11]. The contents of the EV cargo are dependent on their cell of origin, which in case of a tumor may reveal valuable clinical information and have high potential as useful biomarkers.

Various species of RNA have been found in EVs, including microRNA (miRNA), long non-coding RNA (lncRNA), and protein-coding mRNA. All of these RNA types have been proposed as diagnostic or prognostic biomarkers for RCC [12–16]. The distribution of RNA types in EVs demonstrates a high heterogeneity when comparing various biofluids, organ types, and isolation methods [17–19]. In our previous work, we determined mRNA expression profiles from prostate cancer EVs [20]. This strategy allows the simultaneous mapping of hundreds of low-abundance cancer-related mRNA in EVs.

The EV proteome includes all cytosolic proteins within EVs and the transmembrane or GPI-anchored proteins localized at the bilipid membrane [21, 22]. A subset of EV proteins is frequently identified in EVs regardless of origin or isolation methods and is, therefore, used to demonstrate presence of EVs [23]. Other proteins in EVs are specific to cell or tissue of origin and represent potential EV protein biomarkers [21]. Utilizing high-sensitive mass spectrometry (LC/MS), thousands of proteins can be identified [24]. Previously, a multi-omics approach has been used to simultaneously assess protein-expression and gene-expression on RCC tissue [25]. However, in EVs derived from RCC cells, these techniques have not yet been combined.

CCM is frequently utilized by EV researchers due to availability and reproducibility [11, 26]. Because of the known origin of the cell line, the disease specificity of CCM EVs is high, providing an opportunity to identify cancer-specific EV biomarker candidates. Compared with patient samples such as plasma [27], serum-free CCM contains relatively low contamination following EV isolation. The role of EVs in RCC has been studied in CCM [12–14, 21, 24], but no benign kidney cells were included to enable specific biomarker discovery. In this study, we analyze EV mRNA and protein cargo of ccRCC cells (786-O, 769-P, and Caki1), pRCC cells (ACHN and Caki2), and immortalized benign epithelial kidney cells (HK2 and RPTEC/TERT1) [28]. Using this multi-omics approach, we aimed to select candidate diagnostic RCC biomarkers to distinguish benign versus cancer and prognostic biomarkers to distinguish ccRCC and pRCC.

## Results

### Characterization of EVs released from RCC and immortalized benign epithelial kidney cell lines

**Fig. 1:**
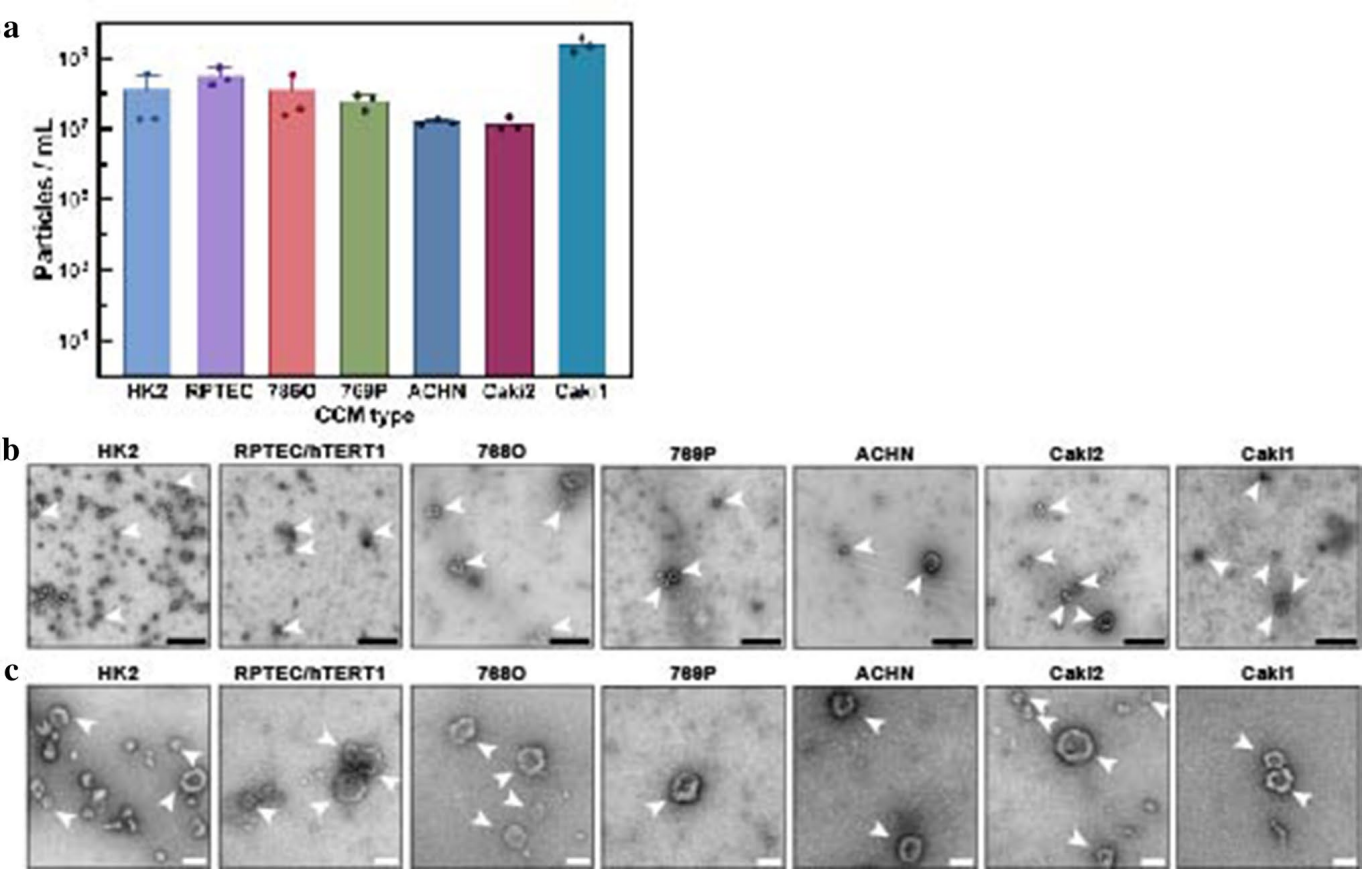
EV characterization by NanoFCM and TEM. **a** Total particle concentrations by cell number at moment of CCM harvest demonstrate number of secreted EVs per million cells. Mean + SD were calculated of three replicates. **b** TEM images confirm the presence of negative-stained EVs, seen as cup-shaped vesicles in widefield view. Black scale bar represents 500 nm. White arrows indicate small EVs. **c** close-up TEM images of kidney EVs. White scale bar represents 100 nm. White arrows indicate small EVs

EVs were isolated from CCM of ccRCC cells (786-O, 769-P, and Caki1), pRCC cells (ACHN and Caki2), and immortalized benign epithelial kidney cells (HK2 and RPTEC/TERT1). The number of isolated EVs per mL CCM as measured by nanoFCM varied per cell type (Figs. 1a, S2). When corrected for the total cell number at harvest, ACHN released the most EVs (1.4 × 10^7^ EVs/million cells) and Caki1 secreted the least (5.6 × 10^4^ EV/million cells) (Fig. 1a). The particle size-distribution was also measured (Fig. S2). The mean particle size for benign kidney EVs was 44.5 ± 1 nm, for ccRCC EVs was 59.5 ± 9 nm, and for pRCC EVs was 51.6 ± 6 nm. EVs were negatively stained and imaged by TEM, which demonstrated intact membranous vesicles for all cell types recognizable by cup-shape on wide-field and close-up (Fig. 1b, c).

**Fig. 2:**
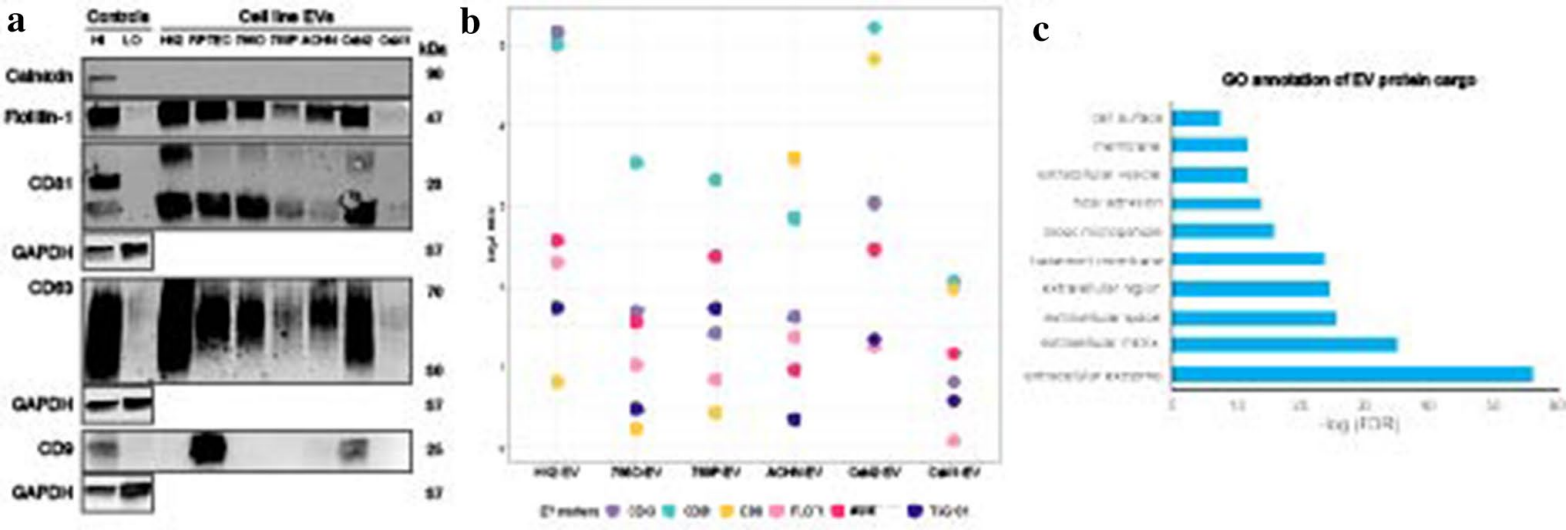
Protein analyses of kidney EVs by western blot and mass spectrometry. **a** Western blots of lysed kidney EVs demonstrate presence of flotillin-1, CD63, and CD81 and absence of cellular debris marker calnexin. Control samples were MCF7 membrane (HI) and cytosolic protein (LO) fractions. **b** Bubble plot of EV protein expression across kidney EVs demonstrates a similar pattern of EV protein expression as seen on the western blots. For each marker, log_2_ ratios were calculated by normalization to the reference channel (pooled cellular reference). **c** Functional Gene Ontology (GO) annotation of the EV-associated proteins commonly identified across six renal cell lines

EVs were analyzed for presence of established EV markers (FLOT1, CD81, CD63, and CD9) and the non-EV cellular marker calnexin (Fig. 2a). Calnexin was negative in all EV samples, indicating absence of ER-protein, a frequently detected non-EV contaminant. While relative abundance of EV markers varied across samples, at least three EV-enriched markers were present in all samples, confirming presence of EVs. Flotillin-1 was highly abundant in EVs from HK2, Caki2, RPTEC, 786-O, and ACHN, while it was of lower abundance in 769-P and very low in Caki1. CD81 was highly abundant in HK2, Caki2, 768O, RPTEC, and 769-P, while low in ACHN and very low in Caki1. CD63 was the highest in HK2 and Caki2, followed by RPTEC, 786-O, and ACHN. 769-P and Caki1 had low CD63 abundance. CD9 was only detected in EVs from RPTEC, Caki2, and ACHN EVs.

### Spatial proteomics confirms presence of EV protein markers

While western blot is a standard in the EV field, detection of positive EV markers is limited by sensitivity and sample input. To robustly characterize the protein cargo of CCM kidney EVs, a previously developed spatial proteomic approach [29] to examine the differential abundance of proteins was adapted across three sample preparations: EV, cell lysate, and debris (“2 k”) pellet. Following sample lysis and proteolytic digestion, the peptides from six renal cell lines (786-O, 769-P, ACHN, CAKI2, CAKI1, and HK2) were subjected to tandem-mass-tagging isobaric labeling followed by tandem mass spectrometry analysis. A pooled reference channel composed of a mix of cell lysates from each of the individual cell lines was included to link the two TMT-plexes (Fig. S1c). In total, 1,967 proteins were identified with quantification information in the combined search of both TMT-plexes, including 1726 proteins quantified across the two TMT-plexes (Table S1).

The differential abundance of classic EV markers FLOT1, CD81, CD63, and CD9 were also tested by western blot, as well as additional EV markers ALIX and TSG101 in EV fractions relative to cell lysate (Fig. 2b) [22]. Using a threshold cutoff of FC > 1.5 (log_2_ FC > 0.58), all the EV markers were found to be elevated in EVs from HK2, 786-O, and CAKI2 renal cell lines and four of the six markers to be increased in 769-P, ACHN, and CAKI1 EVs. Whereas CD81 and CD63 had increased abundance in all the EV fractions relative to the cell lysate, ALIX, CD9, TSG101, and FLOT1, while robustly detected, did not always display increased abundance in the EV fractions relative to the cell lysates. This reflects the variable abundance of these proteins in the individual renal cell lines and EV fractions, as well as the potential variable distribution of EV sub-populations [30, 31]. While negative by western blot, CD9 was detected by mass spectrometry in EV samples from CCM of HK2, 786-O, 769-P, ACHN, and CAKI1. CD81, CD63, and Flot1 abundance was the lowest in Caki1, indicating that while the EV proteins were present in the Caki1 sample, they were likely at too low level to be detected by western blot.

In addition to positive EV markers, the differential abundance of several proteins classically considered to be negative in EVs was also evaluated, including CANX (also measured by western blot), HSP90B1, and CYC1 [22]. Using a threshold cutoff of FC > 1.5 (log_2_ FC > 0.58) between the cell lysate and EV fraction, CANX was found to be the only negative EV marker decreased in all EV samples, following the same expression pattern as shown by western blot, while HSP90B1 was decreased in EVs from five out of the six cell lines.

Finally, to specifically assess the enrichment of EV-specific proteins (i.e., those uniquely present or highly enriched in the EV fraction and absent or depleted in the cell lysate or 2 k debris fraction), proteins with high abundance in EV vs cell lysate and/or in EV vs 2 k debris fraction (log_2_ FC > 0.58) were considered. Following these criteria, 1279 proteins were annotated that were EV-associated in at least one cell line, 420 proteins in four, and 284 proteins in five cell lines (Table S2). 186 proteins were EV-associated in all six cell line sample sets. Using the bioinformatic tool DAVID [32, 33], gene ontology (GO) analysis of the 186 EV-specific proteins identified in all samples and found enrichment for cell component GO annotations “extracellular exosome,” “extracellular matrix,” “focal adhesion,” and “extracellular vesicle” (FDR < 0.05; Fig. 2c, Table S3). The biological process GO terms “extracellular matrix organization,” “cell adhesion,” and “platelet degranulation” were the most prominent biological process annotations for the EV-associated proteins on our dataset (FDR < 0.05; Table S4).

The quantitation information of the cell lysates from the individual cell lines was leveraged to determine the concordance of protein abundance patterns in the cellular and EV fractions. Focusing on the differential abundance of proteins in RCC cell lines and the HK2 cell line (Tables S5 and S6), concordant trends (displaying either increased abundance or decreased abundance) including 76 proteins in ACHN cells/EVs, 59 proteins in CAKI2 cells/EVs, 47 proteins in 786-O cells/EVs, 70 proteins in 769-P cells/EVs, and 39 proteins in CAKI1 cells/EVs were observed (Table S6). These results provide some evidence of EVs reflecting the molecular characteristics of their respective parental cells [34].

Overall, these results confirm that the SEC approach was successful for enriching EVs, and subsequently EV-associated proteins, while reducing the abundance of several proteins commonly categorized as negative markers of EVs.

### Consensus proteomic signature of RCC EV

To better understand the potential biological impact of RCC-derived EVs, the renal cell lines were grouped based on their respective histology: pRCC (ACHN and CAKI2), ccRCC (786-O, 769-P, and CAKI1), and a single benign cell line (HK2) [28]. Using a FC > 1.5 (log_2_ FC > 0.58) threshold, the differential abundance of EV-associated proteins between each of these groups was examined. Notably, direct pair-wise comparison of the cell line-derived EVs revealed a high degree of heterogeneity in protein abundance, emphasizing the heterogeneity of EV cargo itself, even within a single histology type (Tables S5 and S7).

On comparing pRCC EV cargo (n = 2 cell lines) to benign (n = 1 cell line), 31 proteins were found to be increased and 88 proteins were decreased in abundance in EVs from pRCC cell lines compared to EVs from the HK2 benign cell line (Table S5). Using the bioinformatic tool WebGestalt [35, 36] for pathway analysis revealed disparate signaling-related proteins in each of the pRCC EVs, including NOTCH-regulating proteins increased in abundance in EVs derived from ACHN cells, and MET signaling-regulating proteins increased in abundance in the EVs derived from CAKI2 cells (FDR < 0.05; Table S8). Commonly enriched proteins increased in abundance in pRCC EVs were associated with the proteasome degradation (Table S9). More overlaps were observed in the annotated pathways for the proteins increased in HK2 EVs relative to pRCC EVs, including extracellular matrix organization, focal adhesion, endocytosis, and vesicle trafficking (Tables S8 and S9).

Analysis of the differential abundance of proteins derived from EV released from ccRCC cell lines (n = 3 cell lines) and the benign HK2 cell line revealed that 93 proteins were decreased and 34 were increased in the EVs derived from all three ccRCC cell (Table S5). Cellular processes associated with CCT/TriC protein folding and metabolism were increased in ccRCC EVs relative to HK2-derived EVs, whereas proteins associated with neutrophil degranulation and ERBB2 signaling were decreased in ccRCC EVs (FDR < 0.05; Tables S8 and S9).

Finally, the differential abundance of proteins in EV derived from pRCC cell lines (n = 2 cell lines) and ccRCC cell lines (n = 3 cell lines) was assessed. Overall, there were six differentially abundant EV proteins between ccRCC and pRCC: five proteins were increased in EVs from all pRCC cell lines assessed relative to all ccRCC EVs (EDIL3, GC, HBA1, LTF, OLFML2B), while only one protein, HTRA1, was increased in all ccRCC-derived EV samples relative to pRCC EVs (Table S7). Pathway analysis of the proteins found to be differentially abundant between the pRCC EVs and ccRCC EVs revealed disparate pathways in each of the individual RCC EV comparisons (Tables S10 and S11), albeit with ECM–receptor interaction and focal adhesion being commonly annotated across multiple comparisons.

To specifically identify candidate ccRCC or pRCC EV cargo protein biomarkers, the proteins enriched in EV samples were assessed, regardless of relative abundance. To be included in the analysis, a protein must be detected in the EV preparation in all cell lines of the pathological type (i.e., for ccRCC, 786O, 769P, and CAKI1; for pRCC, ACHN, and CAKI2; for benign, there is a single cell line, EVs from the single cell line HK2). 181 proteins were detected in EVs released from all renal cell lines: the benign epithelial kidney cell lines, both pRCC cell lines, and three ccRCC cell lines. While a large number of proteins were enriched in EV from the immortalized benign epithelial cell line, fewer were specifically enriched in RCC EVs. 34 proteins were enriched in pRCC EVs alone, while 20 were uniquely enriched in EV released from ccRCC cells (Fig. 4a, Table S12). These protein sets represent candidate EV biomarkers to discriminate both RCC from benign renal cells as well as discriminate RCC subtype.

### The consensus ccRCC EV mRNA cargo signature

**Fig. 3:**
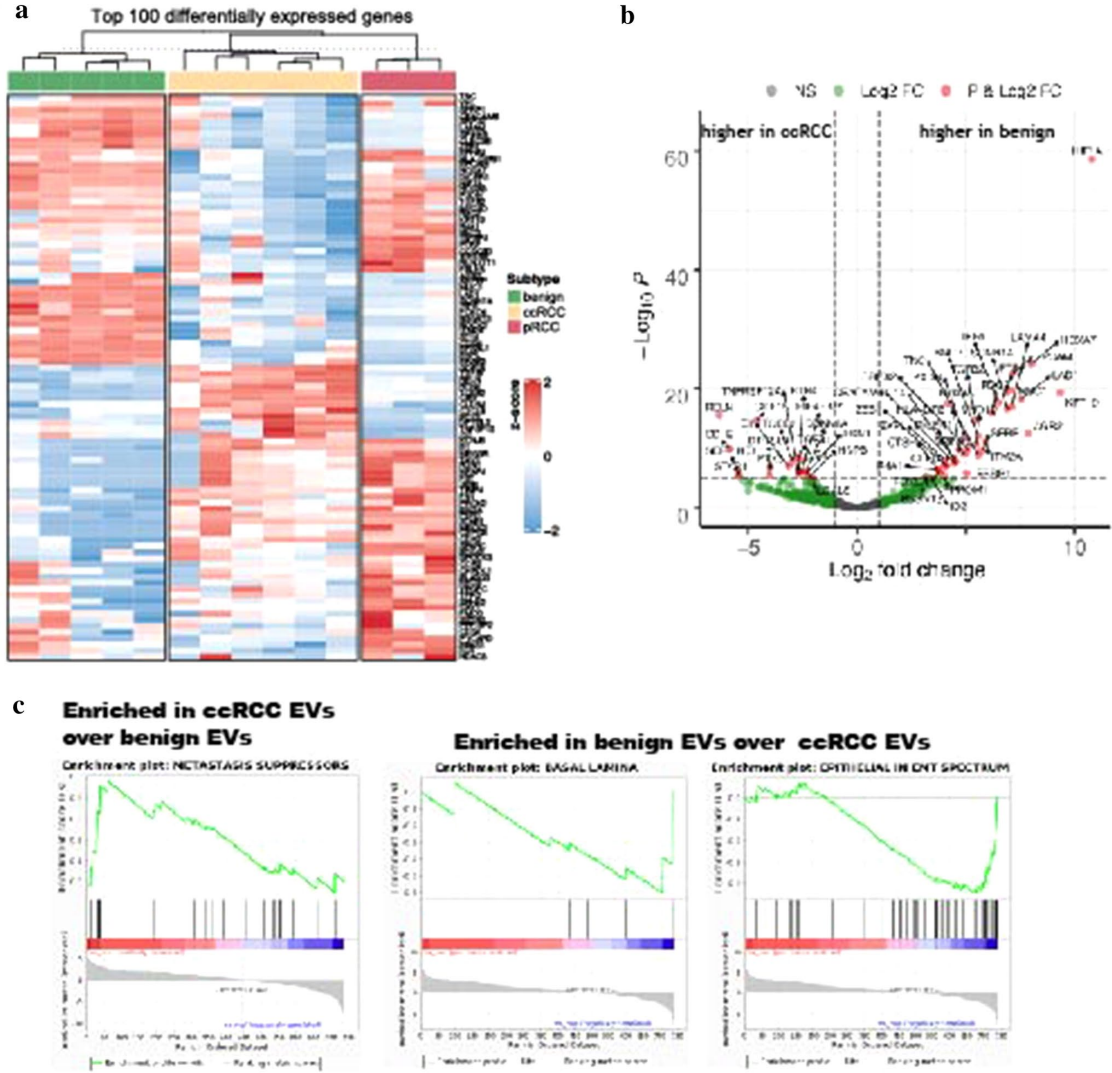
Comparison of the mRNA abundance in benign kidney EVs, ccRCC EVs, and pRCC EVs. Heatmap of top 100 differentially expressed genes using unsupervised clustering demonstrates unique expression patterns for EVs from each subtype (benign, ccRCC, and pRCC). Upregulated genes are in red and downregulated in blue. **c.** Volcano plots demonstrating fold changes of differential gene expression between ccRCC EVs and benign kidney EVs (Data are reported as *x*-axis = log_2_ fold change, *y*-axis = *p* value, dashed lines represent p-value cutoffs of 10e−6, 10e−8, and 10e−5). **d** GSEA plots of Nanostring gene sets enriched in ccRCC or benign EVs (Significance determined as *p* < 0.05. FDR < 0.25)

Total RNA from EVs isolated from seven cell lines (immortalized benign renal cells RPTEC and HK2; ccRCC 786O, 769P, and CAKI1; pRCC ACHN and CAKI2) were run in triplicate using the nCounter PanCancer Progression Panel and data were normalized by total RNA content. Following quality control, 1 replicate of HK2 was excluded due to a technical hybridization error and samples CAKI1 and CAKI2 were excluded due to insufficient RNA input. Of the 770 mRNA transcripts assessed, 461 were detected in one or more samples of CCM EVs, and 159 were present in all five cell line CCM EVs (Table S14). Unsupervised hierarchical clustering of the top 100 most differentially abundant mRNA transcripts indicated that benign renal, ccRCC, and pRCC cell line EVs had distinct mRNA cargos (Fig. 3a). Analysis after quality control was limited to a single pRCC sample, not allowing further analyses assessing pRCC-specific mRNA cargo: such research will be the focus of future work.

Differential gene expression comparing EVs released from ccRCC cell lines and benign renal cell lines demonstrated that *RELN*, *CDH2*, *SERPINE1*,* STAB1*, *VCAM1*, *KCNJ8*, *SRGNR*, *EREG*, *COL6A3*, and *GDF15* were the 10 most enriched genes in RCC EVs, while *HIF1A*, *KRT19*, *HOXA7*, *AGR2*, *LAD1*, *EPCAM*, *LAMA4*, *MUC1*, *SCNN1A,* and *PTRF* were the 10 most enriched genes in benign EVs (Fig. 3b, Table S13). To start investigating the possible biologic functions of EVs released from cancer vs benign renal cells, GSEA was utilized to assess the NanoString-defined gene sets related to cancer progression. The NanoString gene set Metastasis Suppressors was significantly enriched in ccRCC EVs (Fig. 3c). Conversely, the two NanoString gene sets Epithelial in EMT spectrum and Basal Lamina were significantly enriched in benign EVs (Fig. 3c).

**Fig. 4:**
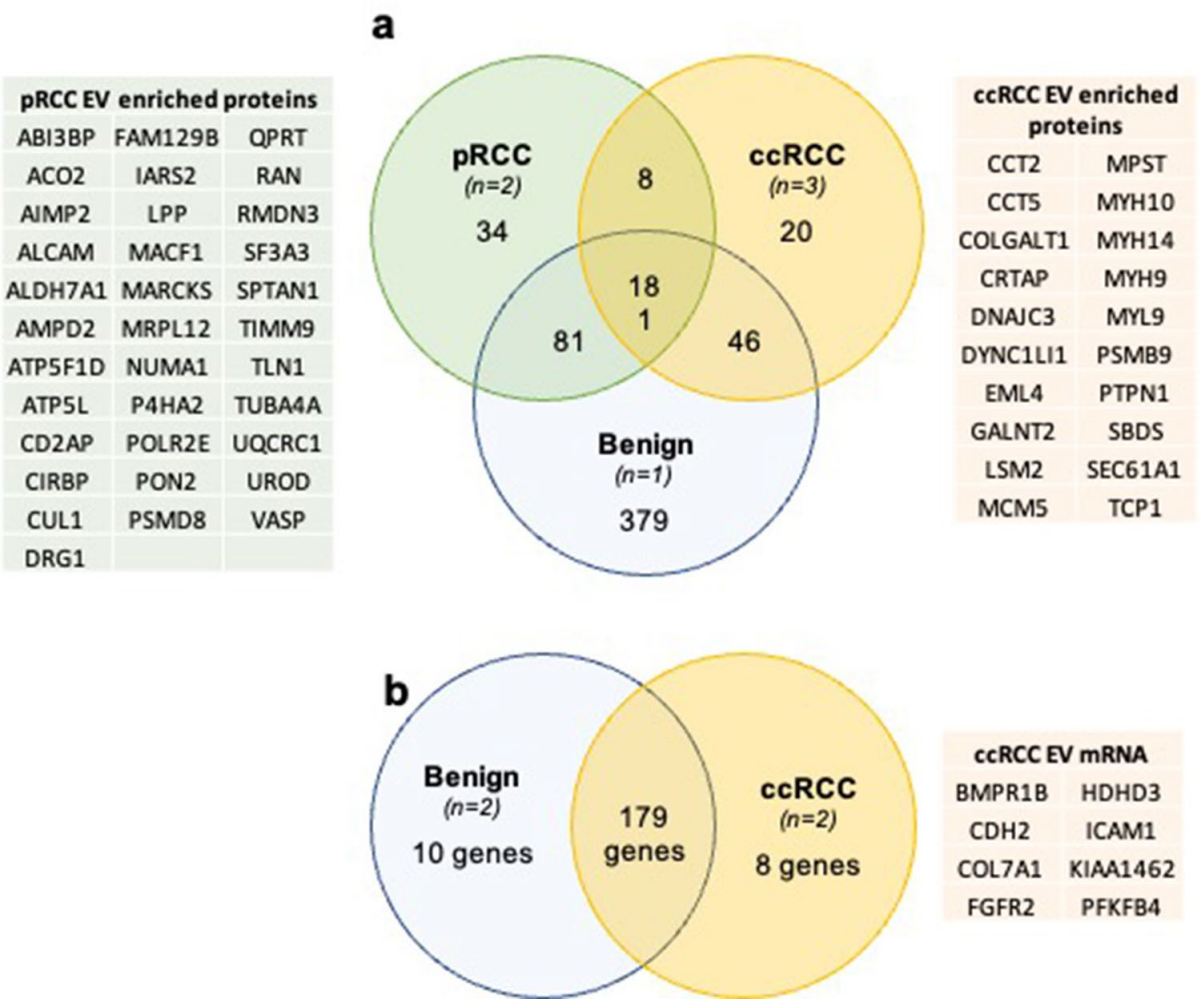
Candidate EV biomarkers for ccRCC and pRCC. **a** Venn diagram demonstrating number of common and unique proteins detected in EVs from benign renal, ccRCC, and pRCC cell lines. Associated tables list candidate EV protein biomarkers for ccRCC and pRCC. **b** Venn diagram demonstrating number of common and unique mRNAs detected in EVs from benign renal and ccRCC cell lines. Table lists candidate EV mRNA biomarkers for ccRCC

To identify candidate ccRCC mRNA EV cargo biomarkers, mRNA transcripts found in benign renal cell EVs and ccRCC EVs were directly compared, regardless of abundance. To be scored that the mRNA had to be detected in the majority of replicates per cell line and present/absent in both cell lines per type. 170 mRNA transcripts were present in EVs released from both the benign epithelial renal cell lines and the ccRCC cell lines (Table S14). Ten mRNAs were unique to benign epithelial kidney EVs and absent in EVs released from ccRCC: *EPCAM*,* PRKCZ*,* PXDN*,* CXADR*,* EPS8L1*,* HOXA7*,* LAD1*,* MYO1D*,* ROCK2*, and *SLC35A3*. Eight mRNAs were found only in ccRCC: *CDH2*, *COL7A1*, *FGFR2*, *BMPR1B*, *HDHD3, ICAM1*, *KIAA1462*, and *PFKFB4* (Fig. 4b).

## Discussion

In this study, a whole proteomic approach and targeted mRNA profiling were leveraged to identify the consensus protein and mRNA signatures of RCC cell lines for the selection of potential biomarkers and to assess biological function of kidney cell line EV cargo. In the literature, only few studies focus on the identity of cargo from EVs derived directly from RCC cells. Jingushi et al. found presence of the LAIR1 protein in RCC tissue as well as in 786-O, Caki1, and Caki2 whole cell lysates and their released EVs [16]. The *LAIR1* mRNA was higher in RNA extracted from the tissue, but measurement of the mRNA transcript directly in EVs was not performed. Qu et al. studied abundance of the long noncoding RNA lncARSR in 786-O and ACHN cells and its sunitinib resistance-promoting effect in xenograft models [14]. De Palma et al. identified a three-mRNA signature *GSTA1, CEBPA, PCBD1* in urinary EVs [15]. Raimondo et al. used MS to identify proteins in pooled urinary EVs from RCC patients compared with urine from no-cancer controls. They selected 10 candidate protein RCC biomarkers in urinary EVs, such as MMP-9, PODXL, DKK4, and CAIX [37]. Jingushi et al. have found the AZU1 protein, which is related to increased extravasation by vascular permeabilization, in EVs from 786O, Caki1, Caki2, and ACHN [24].

EV mRNA data revealed significantly lower mRNA levels of *VHL* in ccRCC EVs as compared to benign EVs in concordance with TCGA data reported by Linehan et al. [38, 39]. Genes for other subunits of the complex (*TCEB1*, *TCEB2*, and *RBX1*) were significantly more abundant in ccRCC EVs compared with benign EVs, perhaps in response to reduced function of the complex due to loss of VHL. The VHL-Elongin BC-complex stabilizes the HIF1A protein which then degrades. With loss of VHL, HIF1A degradation will be less and this could lead to an increased hypoxia response. The *HIF1a*-mRNA was significantly higher in both benign and pRCC EVs compared with ccRCC EVs (Fig. 3b). The hypoxia-response genes *PDK1, VEGF-A,* and *VEGF-C* were significantly more abundant in ccRCC EVs compared with benign EVs. *SETD2*, a chromatin remodeling gene located on chromosome 3p, displayed higher mRNA abundance in benign EVs compared with ccRCC EVs, potentially indicative of a somatic mutation. Loss of *CDKN2A* in the mTOR pathway is associated with ccRCC and mRNA levels were significantly lower in ccRCC compared with benign EVs. *TP53* mRNA was significantly higher in RCC EVs compared with benign EVs.

Brannon et al. studied two mRNA signatures, ccA and ccB, in tissue microarrays of ccRCC. The pattern of ccB was associated with a worse prognosis [39]. Although different subgroups of ccRCC were not studied here, significant differences were found in the angiogenesis-related genes *FLT1, FLT4,* and *KDR* between cancer and benign EVs as well as ccRCC EVs and pRCC EVs, which fits more with a lower aggressive ccA phenotype. *VEGF-B* and *BAI1* did not differ significantly. On comparing the EMT-genes in the ccA and ccB signatures with this study EV data, ccB-associated genes were significantly more abundant in benign EVs (SLP1, TNC, and MMP12), and COL6A1 was more abundant in ccRCC EVs. *CDH2* and *VIM*, higher expressed in ccA phenotype, were significantly more abundant in ccRCC EVs than in benign EVs.

The spatial proteomic approach to robustly characterize the protein cargo of renal cell derived EVs identified over 1900 proteins including well-known EV markers, as well as those considered to be negative markers of EVs. Rather than rely solely on protein identification for classifying a protein as EV cargo, relative protein abundance was utilized as a primary filtering criterion [29, 40]. While it may be inaccurate to characterize proteins that did not meet this filtering criterion as “non-EV proteins,” proteins can be identified that show reduced abundance in renal EVs relative to renal cells and depletion fractions (e.g., 2 k pellet). These would likely be considered as potential contaminants of the EV fraction.

Of potential interest to the EV field is the identification of a consensus renal EV proteome, which includes proteins that are known to be enriched in EVs, including known EV markers, as well as proteins anticipated to be EV cargo such as those associated with the extracellular matrix, cellular membrane, and endosome/receptor internalization. In addition, proteins were identified that canonically belong to intracellular compartments not usually associated with EVs, including nuclear-associated proteins (H2BFS DIP2B, KPNA6) and mitochondria-associated proteins (PGAM5, ACADM, NDUFS1), illustrating that the passive and active mechanisms involved in EV protein routing have yet to be fully elucidated. While this report may be the first to annotate a consensus renal EV proteome, it does not offer any insight into whether the proteins identified can be applicable for defining tissue-specific EV features, warranting a more comprehensive analysis of EV protein cargo from a variety of tissue models.

Evaluation of the differential abundance of EV cargo proteins revealed cellular pathways commonly annotated and associated with EVs, including ECM–receptor interaction and focal adhesion, albeit with variable abundance when comparing pRCC, ccRCC, and HK2 cell-derived EVs relative to one another. These results support the notion that these protein classes are routed into EVs as a reflection of their respective biological function. Additional studies are needed to delineate the disparate impact these cargo profiles may have on EV internalization by recipient cells and subsequent cellular phenotypes. Proteins were identified that were enriched in RCC EVs relative to HK2 EVs, including TNFAIP6, whose mRNA transcript has been shown to be a potential ccRCC biomarker [41], and VCAN, which has been shown to have reduced plasma levels following nephrectomy [42]. Inversely, proteins such as SCAMP3, whose mRNA transcript abundance was found to be increased in normal tissues relative to RCC tissues [43], and STUB1, which is considered a tumor suppressor in pancreatic cancer [44], were enriched in HK2 EVs relative to RCC EVs. Several proteins were different between pRCC and ccRCC EVs including CCT complex-related proteins (CCT2, CCT5, and TCIB). The CCT complex has a role in stabilizing VHL prior to interaction with the VCB-Cul2 ubiquitin ligase complex and subsequent ubiquitination of HIF1/2α substrates [45]. VHL inactivation due to chromosomal loss is a nearly universal event in ccRCC [25], and the increased abundance of the CCT complex proteins in ccRCC EVs, in addition to elevated hypoxic-response gene mRNA levels, may be a consequence of loss of VHL expression at the cell level.

This study demonstrates the heterogeneity of RNA and protein signatures of EVs, likely due to biological variation as well as limitations of current experimental technology. While this study is a critical first step to identifying tumor-specific EV biomarkers, it has a number of limitations. First, both the proliferation rates and per-cell release of EVs of these cell lines vary. In this study, similar amounts of cells were plated, and the EV counts were normalized by cell count. Secondly, due to limited sample availability in combination with the high sensitivity of the TMT-MS, we used a single pooled sample for cell line EV sample. This allowed mRNA and protein assessment in the same EV populations. To increase the accuracy of the MS-based analysis, relative protein abundance compared to parental whole cell lysates was utilized to determine which proteins were enriched in the EV fractions. This robust approach, with the end goal of reducing the number of potential false-positive hits, serves as a method for filtering possibly protein contaminants that may be present in the final EV analyte, an especially important consideration for high-sensitivity techniques. However, the filtering criterion employed in this study would exclude any proteins that display low abundance in an EV fraction relative to the parental cell as EV-associated and may not fully account for the myriad of mechanisms of protein routing into EVs that may impact select EV protein cargo abundance. Finally, loading exactly equal amounts of EV RNA was limited by current methods for RNA quantitation [22, 46, 47]. For NanoString analysis, because standard cell housekeeping genes cannot be used, data are normalized by total read depth per sample. While this approach permits the crucial targeted reverse transcription mRNA amplification necessary for very low mRNA input, it also amplifies any inadvertent loading differences.

This study demonstrates the potential power of EV cargo to be utilized as potential diagnostic and prognostic biomarkers. It also highlights the complicated biology of EV cargo and that further examination is needed to understand how and why RNA and protein is packaged into EVs for expulsion from a cell. Future work remains to be done to characterize the benefits to the cell to secrete EV cargo as well as the potential benefits to cells that internalize EVs.

## Materials and methods

### Cell culture

Kidney cell lines were acquired from the American Type Culture Collection (ATCC®). 769-P and 786-O were cultured in RPMI 1640 media (Gibco™). ACHN was cultured in EMEM media (Gibco™). Caki1 and Caki2 were cultured in McCoy’s 5a medium (Gibco™). HK-2 was cultured in DMEM media (Gibco™). These media were all supplemented with 10% (v/v) fetal-bovine serum (FBS) (VWR™), 100 U/mL penicillin, and 0.1 µg/mL streptomycin (Gibco™). RPTEC–hTERT1 (RPTEC) were cultured in DMEM/F12 supplemented with hTERT-immortalized RPTEC growth kit (ATCC) and 0.2% (v/v) Geneticin™ (Gibco™). Cell lines were regularly screened for mycoplasma.

### Conditioned cell culture media

For 769-P, 786-O, ACHN, Caki1, Caki2, and HK-2, 36 million cells were plated in 500 cm^2^ cell-culture dishes. When confluency reached approximately ~ 60% (typically 48 h after plating), the serum-containing media were removed, cultures were washed twice with PBS, and the media were replaced with appropriate media supplemented with 10% (v/v) exosome-depleted FBS, 100 U/mL penicillin, and 0.1 µg/mL streptomycin (Gibco™). For RPTEC/hTERT1, 45 million cells were plated in 500 cm^2^ cell-culture dishes and confluency reached ~ 60% at day 5. The cultures were washed twice with PBS, and media were replaced with serum- and growth-factor-free DMEM/F12 supplemented with 0.2% (v/v) Geneticin™ (Gibco™). For all cell lines, the media were conditioned for 48 h at 37 °C and 5% CO_2_. Cells were counted at time of harvest using the Countess™ II Automated Cell Counter (Invitrogen™). CCM was collected in triplicate for each cell line and EVs isolated from each replicate.

### Pre-cleaning of conditioned cell culture media

After 48 h of incubation, the CCM was harvested and pre-cleaned by differential centrifugation and filtration. The media were centrifuged at 500×*g* for 5 min. Precipitated cells were kept, PBS-washed, lysed in 1 × RIPA buffer (no. R0278-500 ML, MilliporeSigma), and the cell lysates (CL) stored at − 80 °C. The supernatant was kept and centrifuged at 2000×*g* for 20 min. After this step, the 2 k pellet was kept, PBS-washed, and centrifuged again with same parameters. The 2 k pellets were also resuspended in 1 × RIPA buffer and stored at − 80 °C. The supernatant of the 2000×*g* was centrifuged at 10,000×*g*. The remaining supernatant was filtered using a Nalgene™ Rapid-Flow™ bottle top filter with 0.45 µm PES membrane (Thermo Scientific™). All centrifuge steps were performed at 4 °C (Fig. S1a).

### Ultrafiltration and size exclusion chromatography

CCM was thawed and concentrated in preparation for EV isolation by size-exclusion chromatography followed by additional concentration of the EV sample. All three steps were performed according to the manufacturer’s instructions. Briefly, Centricon® Plus-70 centrifugal filter units (10,000 kDa cutoff, MilliporeSigma) were primed with 50 mL of 0.1 N sodium hydroxide, followed by 65 mL of 0.22 µm-filtered PBS. The retentates of the priming buffers were discarded. Then, 50 mL portions of CCM were concentrated to 500 µL, which was loaded into a SEC column (qEVoriginal, 70 nm, IZON Science) that had been primed with 13 mL of 0.22 µm-filtered PBS. SEC-output fractions of 500 µL each were collected. The fractions with highest abundance of EVs but with the least protein contaminants are fractions 7 to 10 according to the product manual and our previous findings [27]. For each replicate, the EV fractions (i.e., 7 to 10) of five SEC column isolations were combined and concentrated from 10 mL to 200 µL. Of this final EV-product for each replicate, 2 µL was used for counting and TEM. The remainder was used for RNA extraction (Fig. S1b).

### Nanoscale flowcytometry

The Flow Nano Analyzer (NanoFCM inc.) was used to assess particle quantity and size-distribution of the EVs sample in accordance with manufacturers’ manual and as we have done previously [27, 48].

### Transmission electron microscopy

EVs were imaged by transmission electron microscopy (TEM) as previously described [48]. Briefly, the samples were diluted five times in PBS, adsorbed to copper grids, and negative stained. The images were acquired using a Philips CM-120 TEM (80 kV).

### Western blotting

EV samples were lysed with 5 × RIPA buffer (no. AKR-191, Cell Biolabs) with added HALT™ Proteinase and Phosphatase Inhibitor Cocktail (ThermoFisher Scientific). Using the manufacturer’s instructions, the Pierce™ BCA Protein Assay was used to quantify protein content of the EV lysates. Equal amounts of protein (5 µg) were used for western blotting, using our methods as published before [27, 48]. Briefly, for the EV markers Flotilin-1, CD81, CD63, and for non-EV-marker Calnexin [22], the protein was separated on a 10% gel under non-reducing denaturing conditions and transferred to a nitrocellulose membrane. The membrane was incubated overnight at 4 °C with the following antibodies: CD63 (1:250, monoclonal mouse TDS63, no. 10628D, ThermoFisher Scientific); CD81 (1:100, monoclonal mouse 1.3.3.22, no. sc-7637, Santa Cruz Biotechnology); Flotillin-1 (1:2500, monoclonal rabbit EPR604, no. ab133497, Abcam); and Calnexin (1:2500, polyclonal rabbit, no. ab22595, Abcam). Secondary antibodies IRDye® 680RD anti-Mouse IgG (1:20,000, no. 92668070, LI-COR Biosciences) and IRDye® 800CW anti-Rabbit IgG (1:15,000, no. 92632211, LI-COR Biosciences) were used. The blots were imaged with the Odyssey® 9120 Infrared Imaging System (LI-COR Biosciences). For assessment of EV marker CD9, we ran equal amounts of protein under reducing and denaturing conditions, transferred to a PVDF membrane and incubated overnight with CD9 (1:1000, monoclonal rabbit D801A, no. 13174, Cell Signaling Technology). Goat anti-rabbit IgG HRP-linked secondary antibody (1:3000, no. 7074, Cell Signaling Technology) was used with Amersham™ ECL™ Prime Western Blotting Detection Reagent (GE Healthcare). The CD9 blot was imaged on with the ChemiDoc™ XRS + System (Bio-Rad Laboratories). For antibody controls, MCF7 cells were purchased from ATCC® and fractionated into a membrane fraction (high EV markers) and cytosolic fraction (low EV markers) using the Mem-PER™ Plus Membrane Protein Extraction Kit (ThermoFisher Scientific). As a loading control of the cytosolic fraction, the blots were stained for GAPDH (1:2000, Cell Signaling Technology, Cat. # 2118) in second instance followed by washes, incubation with the relevant rabbit secondary antibody, and more washes.

### RNA extraction

For extraction of RNA from the EV samples, the miRNeasy micro kit (Qiagen) was used according to manufacturer’s protocol, including the optional on-column digestion with RNase-free DNase I (Qiagen). The RNA was eluted in 20 µL RNase-free water and stored in − 80 °C until use.

### Multiplexed gene expression analysis

For expression analysis the nCounter PanCancer Progression Panel was used with the low RNA input amplification kit (NanoString Technologies), in accordance with the manufacturer’s instructions [20]. Briefly, 4 µL of RNA sample was converted to cDNA before specific primers were used for cDNA amplification in 14 PCR-cycles. The complete product was used as input for hybridization with nCounter probes for 16 h. Loaded cartridges were run on an nCounter Sprint (NanoString Technologies). Gene expression data quality control was analyzed using nSolver Analysis Software 4.0.70 (NanoString Technologies). Seven samples were excluded from further analysis due to low RNA binding density indicated by gene counts that were below background (all 3 replicates from Caki1, Caki2, and one replicate of HK2). To define presence of a particular mRNA cargo in EV released from each sample, an mRNA was considered present in a cell line if it was detected above threshold in 2/3 replicates (in RPTEC, 786O, 769P, and ACHN) or 2/2 replicates (in HK2). The mRNA cargo was scored as present in a particular histological type (i.e., benign or ccRCC) if it was present in 2/2 cell lines for each type (benign: HK2 and RPEC; ccRCC: 786O and 769P). Because pRCC only contained a single cell line, we did not include pRCC in these analyses. Further normalization and analyses were performed using NanoStringNorm package (Version 1.2.1.1). The remaining samples were normalized for negative controls, positive controls, and for total RNA content via global median scaling. DEseq2 (Version 1.30.1) was used for principal component analysis (PCA) and differential expression analyses. Heatmaps of the top 100 differentially expressed genes between the subgroups of renal EVs (benign, ccRCC, and pRCC) were created using unsupervised clustering.

### Gene set enrichment analysis

Gene set enrichment analysis (GSEA) was applied to determine the potential functional pathways associated with the differentially abundant mRNA transcripts in EVs from various origins, i.e., RCC subtype or benign. The analyses were run on the Broad Institute Gene Set Enrichment Analysis website (http://www.genepattern.org) [49, 50]. Thirty-seven pre-defined gene sets were used as the reference sets, which were downloaded from the Nanostring website (http://www.nanostring.com). The log2 fold and median normalized data were ranked. Then, the GSEA algorithm generated an enrichment score, which estimated whether certain gene sets were enriched in one or the other. A gene set with nominal *p*-value (NOM) *p*-value < 0.05 and False Discovery Rate (FDR) q-value < 0.25 was considered as significantly enriched. The GSEA was repeated with a second run using the 50 Hallmark gene sets from the Molecular Signatures Database [51].

### Proteolytic digestion

Equal parts of CCM from each of the biological replicates used for gene expression analysis were combined for downstream proteomic analyses (Fig. S1c). For six of the kidney cell lines (HK2, ACHN, Caki2, 786-O, 769-P, and Caki1), the CCM samples were pooled (equal to 36 million cells at plating). RPTEC was not used for this experiment. EVs from the CCM were enriched by SEC as described above. Sample preparation for global proteomic characterization were performed as previously described, with some modifications [25]. Briefly, cell lysates, 2 K pellet lysates, and EV lysates were clarified by centrifugation at 20,000×*g* for 10 min at 4 °C prior to buffer exchanged into a compatible digestion buffer (8 M urea, 50 mM Tris–HCl, pH 8.0) using an Amicon Ultra 0.5 mL centrifuge filter (Millipore) with a 10 K membrane cut-off. Protein lysates were subjected to reduction with 5 mM 1,4-Dithiothreitol (DTT) for 30 min at RT, followed by alkylation with 10 mM iodoacetamide for 45 min at RT in the dark. Urea concentration was reduced < 2 M using 50 mM Tris–HCl, pH 8.0. Samples were subjected to tandem digestion of Lys-C (Wako Chemicals) at a ratio of enzyme-to-substrate 1:50 for 2 h at RT followed by trypsin (Promega) at a ratio of enzyme-to-substrate 1:50 overnight at RT (room temperature). The generated peptides were acidified to a final concentration of 1% formic acid, subjected to clean-up using C-18 SepPak columns (Waters) and then dried. Desalted peptides were labeled with 10-plex TMT (Tandem Mass Tag) reagents (Thermo Fisher Scientific) following manufacturer’s instructions. Equal aliquots of TMT-labeled peptides were pooled and desalted using C-18 Stage Tips, dried down, and resuspended in 3% ACN, 0.1% formic acid prior to ESI-LC–MS/MS analysis. A pooled reference channel composed of a mix of cell lysates from each of the individual cell lines was included to link the two TMT-plexes.

### Global proteome ESI-LC–MS/MS data acquisition

For global proteomic analysis, ~ 1 µg of peptide was separated using Easy nLC 1200 UHPLC system (Thermo Fisher Scientific) on an in-house packed 20 cm × 75 µm diameter C18 column (1.9 µm Reprosil-Pur C18-AQ beads (Dr. Maisch GmbH); Picofrit 10 μm opening (New Objective)). The column was heated to 50 °C using a column heater (Phoenix-ST). The flow rate was 0.300 μl/min with 0.1% formic acid and 2% acetonitrile in water (A) and 0.1% formic acid, 90% acetonitrile (B). The peptides were separated with a 6–30% B gradient in 84 min and analyzed using the Thermo Fusion Lumos mass spectrometer (Thermo Fisher Scientific). Parameters were as follows: MS1: resolution—60,000, mass range—350 to 1800 m/z, RF Lens—30%, AGC Target—4.0e^5^, Max IT—50 ms, charge state include—2–6, dynamic exclusion—45 s, top 20 ions selected for MS2; MS2: resolution—50,000, high-energy collision dissociation activation energy (HCD)—37, isolation width (m/z)—0.7, AGC Target—2.0e^5^, Max IT—105 ms.

### Proteome data processing and analysis

All LC–MS/MS files were analyzed by MS-PyCloud, a cloud-based proteomic pipeline developed in Johns Hopkins University to perform database search for spectrum assignments [52] using MS-GF + in this study against a UniprotKB Swiss-Prot human protein database (version May 2018; 20,192 reviewed sequences) [53, 54]. A decoy database was used to assess the false discovery rate (FDR) at PSM, peptide, and protein levels [55]. Peptides were searched with two tryptic ends, allowing up to two missed cleavages. Search parameters included 20 ppm precursor tolerance and 0.06 Da fragment ion tolerance, static modification of carbamidomethylation at cysteine (+ 57.02146), TMT-label modification of N-terminus and lysine (+ 229.16293) and variable modifications of oxidation at methionine (+ 15.99491). Filters used for global data analysis included one PSM per peptide and two peptides per protein, with a 1% FDR threshold at the protein level.

To annotate proteins as EV-associated, the following criteria had to be met: (1) proteins with a log2 FC > 0.58 (1.5 FC) between the extracellular vesicle pellet (EV) and the cell lysate (CL) fraction, or (2) proteins with a log2 FC between 0 and 0.58 (1.0–1.5) between the extracellular vesicle pellet (EV) and the cell lysate (CL) fraction and a log2 FC > 0.58 (1.5 FC) between the extracellular vesicle pellet (EV) and the 2 K pellet (2 K). Proteins considered to be significantly increased or decreased between individual EV sample preparations used the criteria of log2 normalized fold change ± 0.58. Proteins categorized as differentially abundant were subjected to overrepresentation enrichment analysis (ORA) using the bioinformatics tool, WebGestalt [35, 36], and mapped to REACTOME and KEGG pathways. GO assignments and ORA results were considered significant if the reported FDR < 0.05.

## Supplementary Information

Below is the link to the electronic supplementary material.

Supplementary file1 (PDF 1017 KB)

Supplementary file2 (XLSX 1064 KB)

## References

[1] Bray F, Ferlay J, Soerjomataram I, Siegel RL, Torre LA, Jemal A (2018). Global cancer statistics 2018: GLOBOCAN estimates of incidence and mortality worldwide for 36 cancers in 185 countries. CA Cancer J Clin.

[2] SEER*Explorer: An interactive website for SEER cancer statistics [Internet]. Surveillance Research Program, National Cancer Institute. https://seer.cancer.gov/explorer/.

[3] Capitanio U, Montorsi F (2016). Renal cancer. The Lancet.

[4] Johnson DC, Vukina J, Smith AB, Meyer AM, Wheeler SB, Kuo TM (2015). Preoperatively misclassified, surgically removed benign renal masses: a systematic review of surgical series and United States population level burden estimate. J Urol.

[5] Welch HG, Skinner JS, Schroeck FR, Zhou W, Black WC (2018). Regional variation of computed tomographic imaging in the United States and the risk of nephrectomy. JAMA Intern Med.

[6] Shuch B, Amin A, Armstrong AJ, Eble JN, Ficarra V, Lopez-Beltran A (2015). Understanding pathologic variants of renal cell carcinoma: distilling therapeutic opportunities from biologic complexity. Eur Urol.

[7] Marconi L, Dabestani S, Lam TB, Hofmann F, Stewart F, Norrie J (2016). Systematic review and meta-analysis of diagnostic accuracy of percutaneous renal tumour biopsy. Eur Urol.

[8] Siegel RL, Miller KD, Jemal A (2020). Cancer statistics, 2020. CA Cancer J Clin.

[9] van Niel G, D'Angelo G, Raposo G (2018). Shedding light on the cell biology of extracellular vesicles. Nat Rev Mol Cell Biol.

[10] Becker A, Thakur BK, Weiss JM, Kim HS, Peinado H, Lyden D (2016). Extracellular vesicles in cancer: cell-to-cell mediators of metastasis. Cancer Cell.

[11] Dong L, Zieren RC, Wang Y, de Reijke TM, Xue W, Pienta KJ (2019). Recent advances in extracellular vesicle research for urological cancers: from technology to application. Biochim Biophys Acta Rev Cancer.

[12] Butz H, Nofech-Mozes R, Ding Q, Khella HWZ, Szabo PM, Jewett M (2016). Exosomal microRNAs are diagnostic biomarkers and can mediate cell-cell communication in renal cell carcinoma. Eur Urol Focus.

[13] Crentsil VC, Liu H, Sellitti DF (2018). Comparison of exosomal microRNAs secreted by 786-O clear cell renal carcinoma cells and HK-2 proximal tubule-derived cells in culture identifies microRNA-205 as a potential biomarker of clear cell renal carcinoma. Oncol Lett.

[14] Qu L, Ding J, Chen C, Wu ZJ, Liu B, Gao Y (2016). Exosome-transmitted lncARSR promotes sunitinib resistance in renal cancer by acting as a competing endogenous RNA. Cancer Cell.

[15] De Palma G, Sallustio F, Curci C, Galleggiante V, Rutigliano M, Serino G (2016). The three-gene signature in urinary extracellular vesicles from patients with clear cell renal cell carcinoma. J Cancer.

[16] Jingushi K, Uemura M, Nakano K, Hayashi Y, Wang C, Ishizuya Y (2019). Leukocyteassociated immunoglobulinlike receptor 1 promotes tumorigenesis in RCC. Oncol Rep.

[17] Ferguson SW, Nguyen J (2016). Exosomes as therapeutics: the implications of molecular composition and exosomal heterogeneity. J Control Release.

[18] Wei Z, Batagov AO, Schinelli S, Wang J, Wang Y, El Fatimy R (2017). Coding and noncoding landscape of extracellular RNA released by human glioma stem cells. Nat Commun.

[19] Li Y, Zhao J, Yu S, Wang Z, He X, Su Y (2019). Extracellular vesicles long RNA sequencing reveals abundant mRNA, circRNA, and lncRNA in human blood as potential biomarkers for cancer diagnosis. Clin Chem.

[20] Dong L, Huang CY, Johnson EJ, Yang L, Zieren RC, Horie K (2021). High-throughput simultaneous mRNA profiling using nCounter technology demonstrates that extracellular vesicles contain different mRNA transcripts than their parental prostate cancer cells. Anal Chem.

[21] Hurwitz SN, Rider MA, Bundy JL, Liu X, Singh RK, Meckes DG (2016). Proteomic profiling of NCI-60 extracellular vesicles uncovers common protein cargo and cancer type-specific biomarkers. Oncotarget.

[22] Thery C, Witwer KW, Aikawa E, Alcaraz MJ, Anderson JD, Andriantsitohaina R (2018). Minimal information for studies of extracellular vesicles 2018 (MISEV2018): a position statement of the International Society for Extracellular Vesicles and update of the MISEV2014 guidelines. J Extracell Vesicles.

[23] Liu DSK, Upton FM, Rees E, Limb C, Jiao LR, Krell J (2020). Size-Exclusion chromatography as a technique for the investigation of novel extracellular vesicles in cancer. Cancers.

[24] Jingushi K, Uemura M, Ohnishi N, Nakata W, Fujita K, Naito T (2018). Extracellular vesicles isolated from human renal cell carcinoma tissues disrupt vascular endothelial cell morphology via azurocidin. Int J Cancer.

[25] Clark DJ, Dhanasekaran SM, Petralia F, Pan J, Song X, Hu Y (2019). Integrated proteogenomic characterization of clear cell renal cell carcinoma. Cell.

[26] Gardiner C, Di Vizio D, Sahoo S, Thery C, Witwer KW, Wauben M (2016). Techniques used for the isolation and characterization of extracellular vesicles: results of a worldwide survey. J Extracell Vesicles.

[27] Dong L, Zieren RC, Horie K, Kim CJ, Mallick E, Jing Y (2021). Comprehensive evaluation of methods for small extracellular vesicles separation from human plasma, urine and cell culture medium. J. Extracell. Vesicles..

[28] Brodaczewska KK, Szczylik C, Fiedorowicz M, Porta C, Czarnecka AM (2016). Choosing the right cell line for renal cell cancer research. Mol Cancer.

[29] Clark DJ, Fondrie WE, Liao Z, Hanson PI, Fulton A, Mao L (2015). Redefining the breast cancer exosome proteome by tandem mass tag quantitative proteomics and multivariate cluster analysis. Anal Chem.

[30] Willms E, Johansson HJ, Mager I, Lee Y, Blomberg KE, Sadik M (2016). Cells release subpopulations of exosomes with distinct molecular and biological properties. Sci Rep.

[31] Crescitelli R, Lasser C, Jang SC, Cvjetkovic A, Malmhall C, Karimi N (2020). Subpopulations of extracellular vesicles from human metastatic melanoma tissue identified by quantitative proteomics after optimized isolation. J Extracell Vesicles.

[32] da Huang W, Sherman BT, Lempicki RA (2009). Systematic and integrative analysis of large gene lists using DAVID bioinformatics resources. Nat Protoc.

[33] da Huang W, Sherman BT, Lempicki RA (2009). Bioinformatics enrichment tools: paths toward the comprehensive functional analysis of large gene lists. Nucleic Acids Res.

[34] Pomatto MAC, Gai C, Bussolati B, Camussi G (2017). Extracellular vesicles in renal pathophysiology. Front Mol Biosci.

[35] Liao Y, Wang J, Jaehnig EJ, Shi Z, Zhang B (2019). WebGestalt 2019: gene set analysis toolkit with revamped UIs and APIs. Nucleic Acids Res.

[36] Wang J, Vasaikar S, Shi Z, Greer M, Zhang B (2017). WebGestalt 2017: a more comprehensive, powerful, flexible and interactive gene set enrichment analysis toolkit. Nucleic Acids Res.

[37] Raimondo F, Morosi L, Corbetta S, Chinello C, Brambilla P, Della Mina P (2013). Differential protein profiling of renal cell carcinoma urinary exosomes. Mol Biosyst.

[38] Linehan WM, Ricketts CJ (2019). The Cancer Genome Atlas of renal cell carcinoma: findings and clinical implications. Nat Rev Urol.

[39] Rowbotham DA, Enfield KS, Martinez VD, Thu KL, Vucic EA, Stewart GL (2014). Multiple components of the VHL tumor suppressor complex are frequently affected by DNA copy number loss in pheochromocytoma. Int J Endocrinol..

[40] Marelli M, Smith JJ, Jung S, Yi E, Nesvizhskii AI, Christmas RH (2004). Quantitative mass spectrometry reveals a role for the GTPase Rho1p in actin organization on the peroxisome membrane. J Cell Biol.

[41] Eikrem OS, Strauss P, Beisland C, Scherer A, Landolt L, Flatberg A (2016). Development and confirmation of potential gene classifiers of human clear cell renal cell carcinoma using next-generation RNA sequencing. Scand J Urol.

[42] Mitsui Y, Shiina H, Kato T, Maekawa S, Hashimoto Y, Shiina M (2017). Versican promotes tumor progression, metastasis and predicts poor prognosis in renal carcinoma. Mol Cancer Res.

[43] Zhou A, Liu H, Tang B (2020). Comprehensive evaluation of endocytosis-associated protein SCAMP3 in hepatocellular carcinoma. Pharmgenom Pers Med.

[44] Gallo LH, Ko J, Donoghue DJ (2017). The importance of regulatory ubiquitination in cancer and metastasis. Cell Cycle.

[45] Hansen WJ, Ohh M, Moslehi J, Kondo K, Kaelin WG, Welch WJ (2002). Diverse effects of mutations in exon II of the von Hippel-Lindau (VHL) tumor suppressor gene on the interaction of pVHL with the cytosolic chaperonin and pVHL-dependent ubiquitin ligase activity. Mol Cell Biol.

[46] Mateescu B, Kowal EJ, van Balkom BW, Bartel S, Bhattacharyya SN, Buzas EI (2017). Obstacles and opportunities in the functional analysis of extracellular vesicle RNA: an ISEV position paper. J Extracell Vesicles.

[47] Chen S, Zhu X, Huang S (2020). Clinical applications of extracellular vesicle long RNAs. Crit Rev Clin Lab Sci.

[48] Zieren RC, Dong L, Pierorazio PM, Pienta KJ, de Reijke TM, Amend SR (2020). Extracellular vesicle isolation from human renal cancer tissue. Med Oncol.

[49] Subramanian A, Tamayo P, Mootha VK, Mukherjee S, Ebert BL, Gillette MA (2005). Gene set enrichment analysis: a knowledge-based approach for interpreting genome-wide expression profiles. Proc Natl Acad Sci USA.

[50] Mootha VK, Lindgren CM, Eriksson KF, Subramanian A, Sihag S, Lehar J (2003). PGC-1alpha-responsive genes involved in oxidative phosphorylation are coordinately downregulated in human diabetes. Nat Genet.

[51] Liberzon A, Birger C, Thorvaldsdottir H, Ghandi M, Mesirov JP, Tamayo P (2015). The molecular signatures database (MSigDB) hallmark gene set collection. Cell Syst.

[52] Chen L, Zhang B, Schnaubelt M, Shah P, Aiyetan P, Chan D (2018). MS-PyCloud: An open-source, cloud computing-based pipeline for LC-MS/MS data analysis. BioRxiv..

[53] Kim S, Gupta N, Pevzner PA (2008). Spectral probabilities and generating functions of tandem mass spectra: A strike against decoy databases. J Proteome Res.

[54] Kim S, Pevzner PA (2014). MS-GF+ makes progress towards a universal database search tool for proteomics. Nat Commun.

[55] Elias JE, Gygi SP (2007). Target-decoy search strategy for increased confidence in large-scale protein identifications by mass spectrometry. Nat Methods.

