# Supplementary material for: Defining candidate mRNA and protein EV biomarkers to discriminate ccRCC and pRCC from non-malignant renal cells in vitro
Source: Med Oncol. 2021 Jul 31;38(9):105. doi: 10.1007/s12032-021-01554-2 (PMC8325656; doi:10.1007/s12032-021-01554-2)
Supplement: Supplementary file 1 — Supplementary file1 (PDF 1017 KB) [file 12032_2021_1554_MOESM1_ESM.pdf]

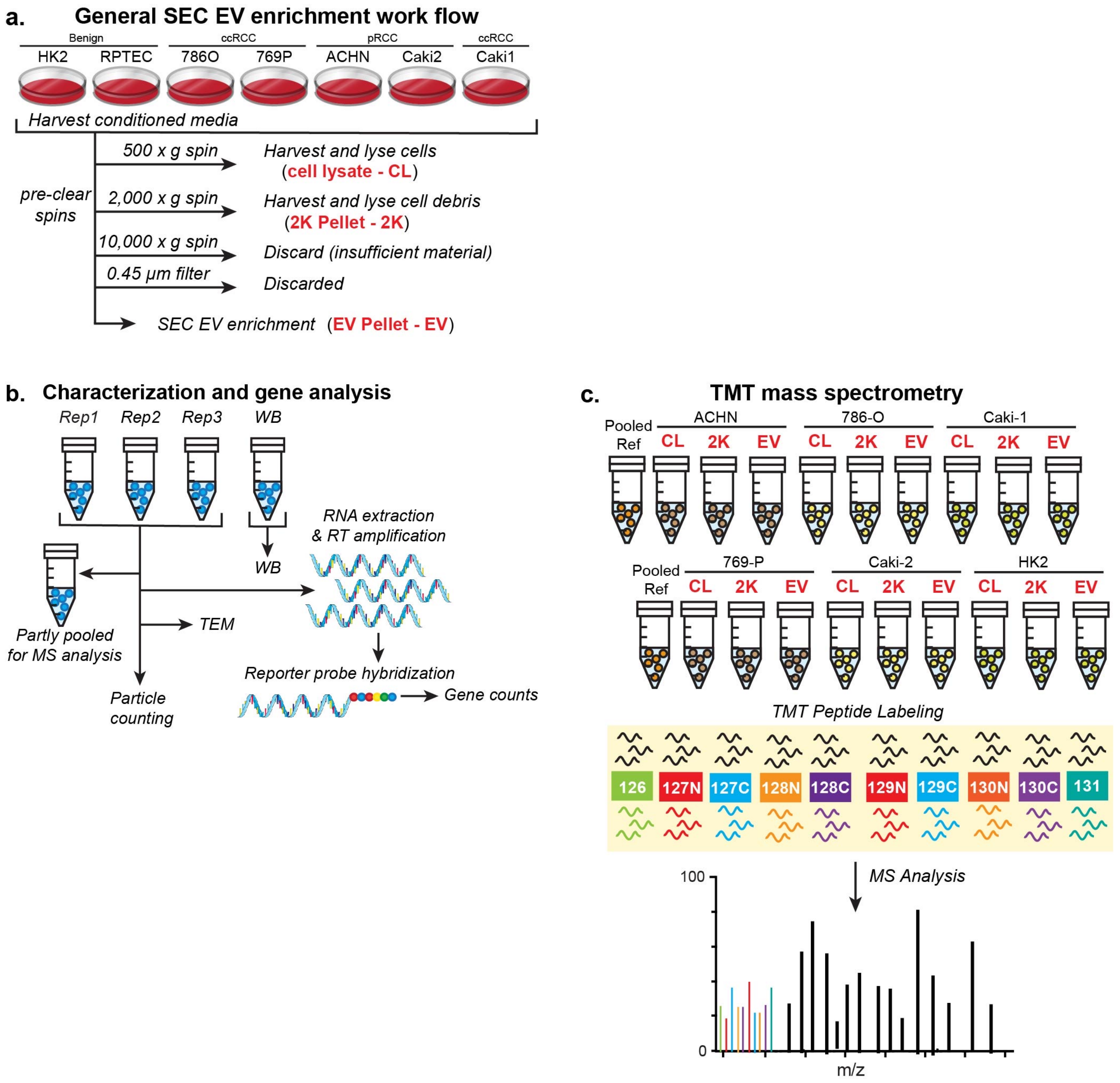

Sup. Fig. S1: **Overall workflow.**

(a) General pre-cleaning steps followed by SEC for EV enrichment of CCM from immortalized benign kidney epithelial cells (HK2 and RPTEC), ccRCC cells (786-O, 769-P, and Caki-1), and pRCC cells (ACHN and Caki-2). (b) Of three biological replicates for a part is pooled to obtain one additional, representative sample for MS analysis. Of the triplicates EVs were counted, TEM images were taken, and RNA was extracted, amplified, labeled with reporter probes for the nCounter gene analysis assay. A separate biological replicate was completely used for protein expression analysis of EV markers by Western Blot. RT – reverse transcriptase. (c) Schematic of proteomic analysis of RCC-derived extracellular vesicles. Three preparations (cell lysate, 2k pellet, and EV pellet) were generated for each individual cell lines were lysed, subjected to proteolytic digestion, TMT labeling, and mass spectrometry analysis. CL – cell lysate, 2k – pellet of 2,000 x G spin, MS – Mass spectrometry, TMT – Tandem Mass Tag

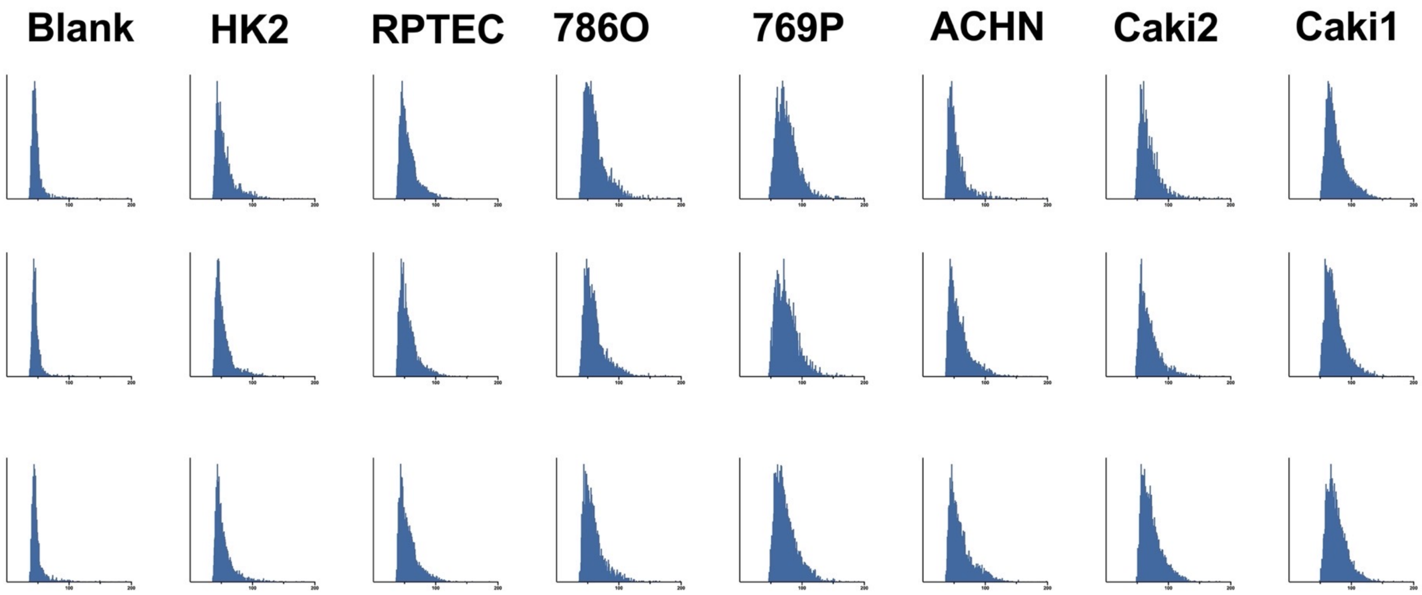

Sup. Fig. S2: Size distribution curves of kidney EVs measured by NanoFCM. For each cell type three replicates are demonstrated and a no-cell CCM control (blank). X-axis is particle diameter [plot range 0-300 nm]. Y-axis is particle concentration with the modus set to 95% of Y-axis range to improve visualization of size distribution on horizontal axis.

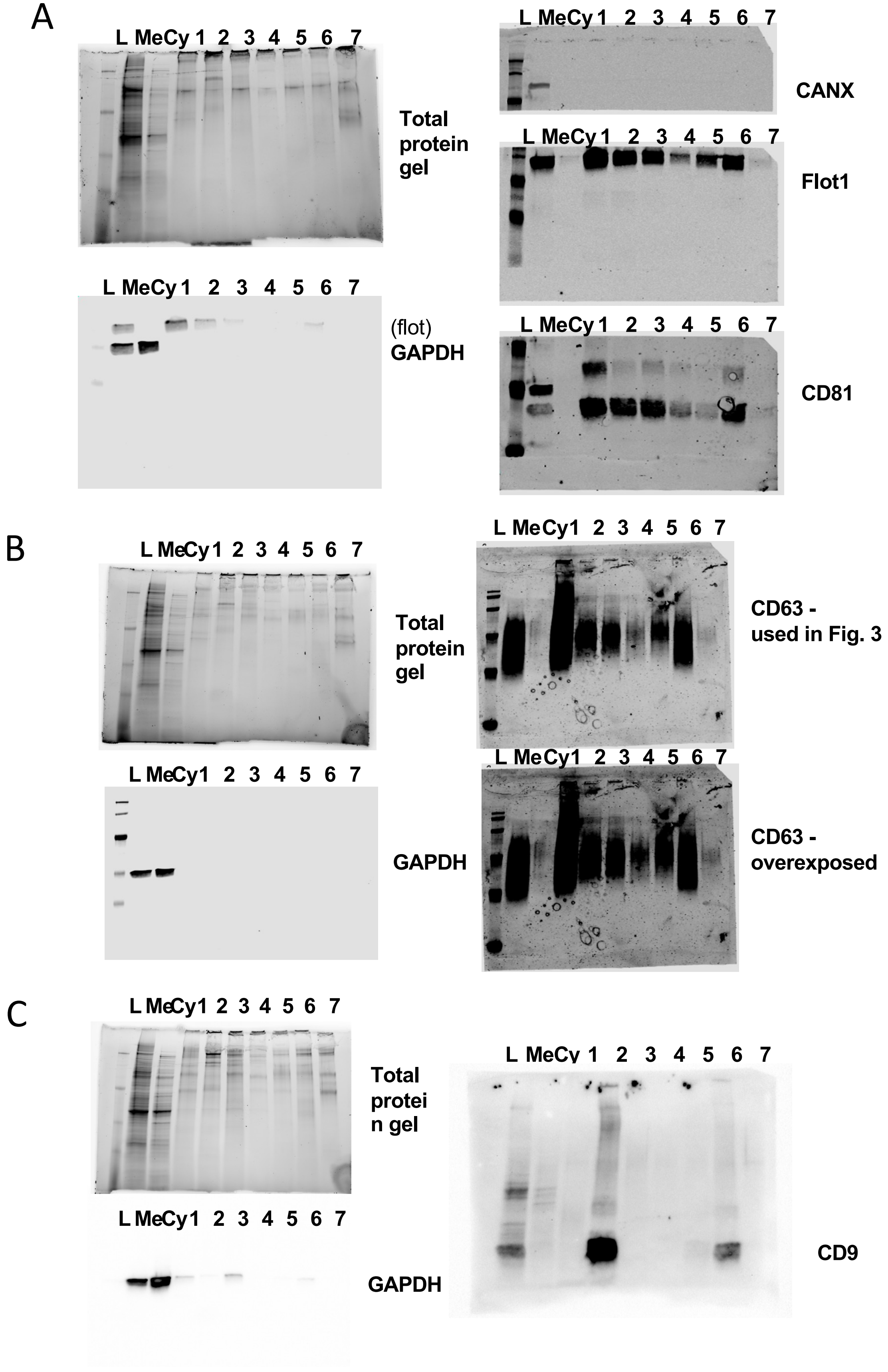

Sup. Fig. S3: Full images of western blots of (A)CANX – Flot1 – CD81 - GAPDH markers under non-reducing conditions. (B) CD63 - GAPDH markers under non-reducing conditions. (C) CD9 - GAPDH markers under reducing conditions. L = ladder; Me = membrane MCF7 fraction; Cy = cytosolic MCF7 fraction; 1 = HK2-EV; 2 = RPTEC-EV; 3 = 786O-EV; 4 = 769P-EV; 5 = ACHN-EV; 6 = Caki2-EV; 7 = Caki1-EV.
